# Competitive Displacement between *Bemisia tabaci* MEAM1 and MED and Evidence for Multiple Invasions of MED

**DOI:** 10.3390/insects11010035

**Published:** 2019-12-31

**Authors:** Xiao-Tian Tang, Li Cai, Yuan Shen, Li-Li Xu, Yu-Zhou Du

**Affiliations:** 1 School of Horticulture and Plant Protection & Institute of Applied Entomology, Yangzhou University, Yangzhou 225009, China; licai988@outlook.com (L.C.); 18061905050@163.com (Y.S.); xulili9012@outlook.com (L.-L.X.); 2 Department of Entomology, Texas A&M University, College Station, TX 77843, USA; tangxt@tamu.edu; 3 Agriculture and Forestry Bureau of Binhu District, Wuxi 214071, China; 4 Joint International Research Laboratory of Agriculture and Agri-Product Safety, the Ministry of Education, Yangzhou University, Yangzhou 225009, China

**Keywords:** *Bemisia tabaci*, invasion, displacement, mechanism, multiple invasion

## Abstract

Despite the severe ecological damage and economic loss caused by invasive species, the factors contributing to successful invasion or displacement remain elusive. The whitefly, *Bemisia tabaci* (Gennadius), is an important invasive agricultural pest worldwide, causing severe damage to numerous crops by feeding or transmitting plant viruses. In this study, we monitored the dynamics of two invasive whitefly cryptic species, Middle East-Asia Minor 1 (MEAM1) and Mediterranean (MED), in Jiangsu, China, from 2005–2016. We found that *B. tabaci* MED quickly established and asserted dominance over MEAM1, resulting in their population displacement in Jiangsu in only three years (from 2005 to 2008). We further investigated the possible mechanisms underlying the successful invasion and competitive displacement from a genetic perspective. Based on sequencing of mitochondrial gene sequences from large numbers of whitefly samples, multiple invasion events of MED were validated by our genetic analyses. MED invaded Jiangsu starting from multiple introduction sites with secondary and/or subsequent invasive events. This may favor their invasion and displacement of MEAM1. This study advances our understanding of the mechanisms that enabled the successful invasion of MED.

## 1. Introduction

Biological invasions are of great concern as they have a pronounced impact on the native ecosystem, biodiversity, and the economy. Displacement events are intimately linked to invasion biology and continue to increase at alarming rates [1]. However, the mechanisms and factors underlying the successful invasion or displacement remain elusive and therefore need to be elucidated. 

The whitefly, *Bemisia tabaci* (Gennadius) (Hemiptera: Aleyrodidae), is a cryptic species complex containing at least 40 morphologically indistinguishable species [2,3,4,5]. Among this species complex, some members are important pests of horticultural and industrial crops and cause large yield loss in agronomic ecosystems worldwide. The Middle East-Asia Minor 1 (MEAM1, commonly known as the B biotype) and the Mediterranean (MED, commonly known as the Q biotype) whiteflies have drawn great attention due to their global invasion and rapid replacement of the native whiteflies [5,6]. MEAM1 entered China in the mid-1990s; however, MED was first detected on ornamental plants in Yunnan in 2003 [7]. In the same year, Chu et al. [8] also detected a few MED individuals in Beijing, Yunnan, and Henan. In 2007, MED individuals were detected from 19 collection sites across 13 provinces, where MED had dominated over 50% of all whitefly samples in 10 out of the 19 collection sites [9]. Shortly after, MED spread rapidly across China [10,11,12,13]. The invasion history of MED in China could be divided into three stages: the early stage (2003), the spread stage (2004–2007), and the unprecedented outbreak (after 2008). Interestingly, MED dominates MEAM1, as well as several indigenous species of whiteflies (e.g., Asia II and China 1) in many regions [11,14]. A few studies have investigated the mechanisms underlying this displacement from both intrinsic and environmental perspectives. It has been proposed that such successful invasions and rapid displacement can be attributed to insect behavior (reproduction or feeding) [15,16], insecticide resistance [17], host plants [18,19,20,21], temperature and relative humidity [21,22], and even plant viruses [23,24]. For instance, Sun et al. [18] found that field populations of MED have lower susceptibility to commonly used insecticides (e.g., imidacloprid) than MEAM1, which may play a major advantageous role in competitive displacement. In addition, host plant was found to be one of the most important factors in the competitive fitness of MED and MEAM1. Chu et al. [21] found that when MEAM1 is reared simultaneously with MED on pepper plants, the MEAM1 population decreased significantly from 66.7% to 13.6% and 3.7% in the first and second generation, respectively. However, few studies have addressed the mechanisms from a genetic perspective. Li et al. [25] identified mitochondrial haplotypes of MED and proposed that the genotypes may be associated with MED’s ability to access the invasion habitat. In addition, genetic traits, such as genetic diversity and genetic structure, could also be involved in successful invasions or colonization of alien organisms [26,27]. For example, Crawford and Whitney [28] found that increased genetic diversity enhanced colonization success for the weedy herb *Arabidopsis thaliana*. 

In Jiangsu province, MEAM1 was initially found in 2001, whereas MED was first detected in 2005, and since, they have spread over most of Jiangsu’s regions in only a few years [29]. In this study, we monitored the dynamics of whitefly cryptic species across the northern, central, and southern parts of Jiangsu from 2005 to 2016. Over the 12 years of monitoring, we found that MED quickly established and largely replaced MEAM1. Because genetic diversity or genetic structure may contribute to successful invasion [26,27], we applied genetic data to decipher any possible underlying mechanisms. The main goals of this study were to: (1) present the invasive history and dynamics of MED and MEAM1 in Jiangsu over a decade; (2) investigate the genetic diversity and population genetic structure of MED and MEAM1; and (3) explore the possible mechanisms underlying the successful invasion of MED and competitive displacement between MED and MEAM1 from a genetic perspective. 

## 2. Materials and Methods 

### 2.1. Sample Collection

**Figure 1:**
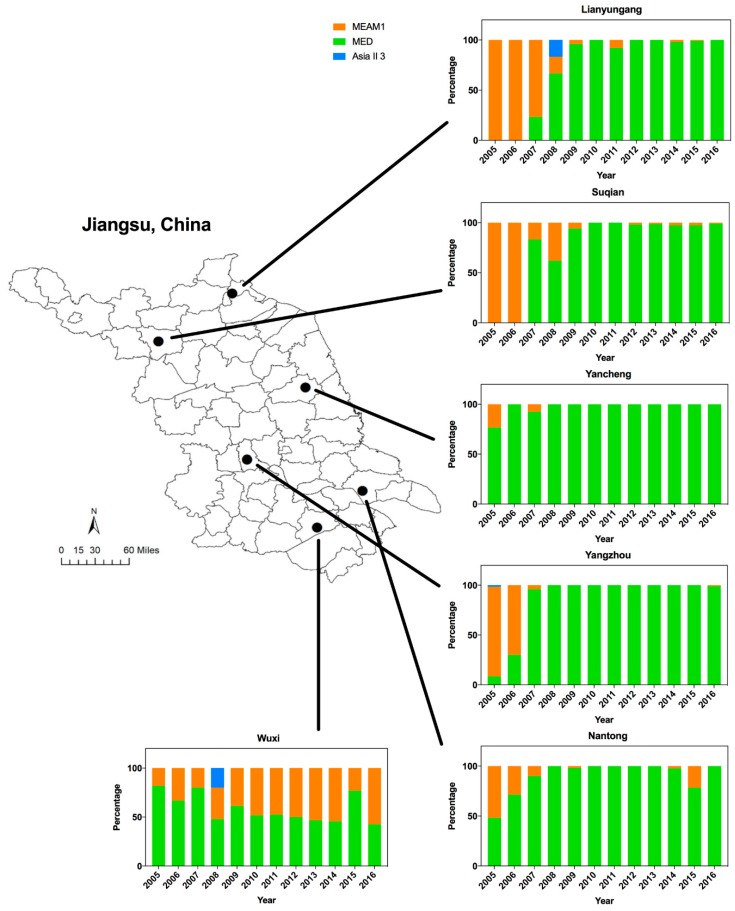
The dynamics of Middle East-Asia Minor 1 (MEAM1), Mediterranean (MED), and Asia II 3 in Jiangsu from 2005 to 2016. The map of Jiangsu was created using Esri’s ArcGIS platform (http://www.esri.com/software/arcgis).

*B. tabaci* samples were collected from greenhouses and cultivated fields across six municipal level administrative units (Lianyungang, Suqian, Yancheng, Yangzhou, Nantong, and Wuxi) in Jiangsu Province from 2005 to 2016 (from August to October) (Figure 1). Collection details, the host plants, and number of sequenced whiteflies are summarized in Table S1. Each collection was obtained by randomly sampling using a self-made aspirator. To identify the cryptic species of whitefly in our collected samples accurately, a total of 4936 *B. tabaci* individuals across each year and each collection site were selected for sequencing of mitochondrial genes. 

### 2.2. DNA Extraction and Amplification

Total DNA was extracted from individual whiteflies following the protocol of Luo et al. [30]. The specific primers F-BQ-880 (5′-TGGAATAGATGTAGATACTC-3′) and R-BQ-1460 (5′-CTTACACCAAGCCTAAATCTTACTA-3′) were self-designed to obtain the mitochondrial Cytochrome Oxidase 1 (CO1) gene fragment. We also tested the non-MEAM1/MED individuals using the universal primers of C1-J-2195 and L2-N-3014 [31]. The Polymerase Chain Reaction (PCR) mixture consisted of 1 U Taq DNA polymerase (Takara, Dalian, China), 5 µL (10×) reaction buffer, corresponding to a final concentration of 3 µL MgCl_2_ (25 mmol/L), 2 µL dNTPs (10 mmol/L), 2 µL forward and reverse primers (20 µmol/L each), and 2 µL of template DNA. The PCR reaction program was initialized at 94 °C for 2 min, followed by 35 cycles of 94 °C for 1 min, 56 °C for 1 min, and 72 °C for 1 min, with a final extension for 5 min at 72 °C. Amplification products were purified and sequenced by IGE Biotechnology Co., Ltd. (Guangzhou, China).

### 2.3. Data Analyses

Sequence fragments of the mtDNA CO1 gene (555 bp) were assembled using ContigExpress to obtain a consensus sequence and then aligned using the Clustal X 1.83 program [32]. Each sequence of the *B. tabaci* sample was blasted against NCBI (http://blast.ncbi.nlm.nih.gov/Blast.cgi) to determine the subspecies/biotype. The sequences in this study were deposited in GenBank (accession numbers: KF142153–KF142157). Each sequence was translated into amino acids to detect whether there was a frameshift mutation or nonsense codon to exclude the pseudogenes. The protein sequences could also be found in GenBank. The haplotype diversity (H) and nucleotide diversity (π) were calculated using DnaSP v. 5.0 [33]. The median joining network of haplotypes was constructed using Network v. 4 [34] and used to infer evolutionary relationships among haplotypes. SAMOVA 2.0 (Spatial Analysis Of Molecular Variance) [35] was used to define the genetic structure of populations with K values ranging from 2 to 6 (for the total six populations of MED) and 100 independent simulated annealing processes. Pairwise *F*-statistic (*F*_ST_) values were calculated using Arlequin v. 3.5 [36] to estimate the degree of genetic differentiation among populations. Sequences were analyzed with other whitefly sequences from NCBI to investigate the phylogenetic status of haplotypes in this study. Both Bayesian Inferences (BI) and Maximum Likelihood (ML) analysis were used. Phylogenetic reconstructions were constructed using the MrBayes program [37] with 3,000,000 generations and with the first 25% being discarded as “burn-in”. The ML tree was constructed in a PHYML online web server [38]. The greenhouse whitefly, *Trialeurodes vaporariorum*, was used as an outgroup. Tree data were visualized and edited using FigTree v. 1.3.1 (http://tree.bio.ed.ac.uk/software/figtree/). Tajima’s *D* and Fu’s *Fs* tests for all populations (pooled) were used to test for neutrality using Arlequin v. 3.5 [36]. Harpending’s Raggedness (HR) index was also calculated in Arlequin v. 3.5. All parameters were evaluated based on 1000 bootstrap replicates. We also investigated the recent population history by estimating the changes in the effective population size over time using a Bayesian skyline plot [39] implemented in the software BEAST Version 2.5.0 [40]. The convergence and output of BEAST were checked and analyzed by TRACER Version 1.7.1 [41].

## 3. Results

### 3.1. Dynamics of Whitefly Cryptic Species in Jiangsu from 2005 to 2016

Analyses of the *B. tabaci* mtCOI sequences showed that among the total collected whiteflies, 21.7% were identified as MEAM1 (invasive), 77.4% as MED (invasive) and 0.9% as Asia II 3 (indigenous). Both MEAM1 and MED were widely distributed across Jiangsu, while Asia II 3 was rarely detected (Figure 1).

The dynamics of whitefly cryptic species has changed dramatically in Jiangsu over the past 12 years. In particular, there was a shift of dominance from MEAM1 to MED from 2005 to 2008 (Figure 1). Overall, the spread of MED began around 2005 (43.3%), accelerated in 2006–2007 (81.5%), was established by 2008 (86.7%), and fluctuated from 86.7% to 92.3% in the following years. Furthermore, MED could largely be detected in Yancheng, Yangzhou, and Wuxi from 2005, whereas MED was not found in Lianyungang and Suqian until 2007. In Yangzhou, few MED individuals could be detected from 2005 to 2006. However, the percentage of MED in Wuxi decreased from 81.7% (2005) to 51.7% (2010) and fluctuated between 42.6 and 77.1% (2010–2016). By combining the results together, MED has displaced MEAM1 and indigenous species in most regions north of Wuxi (Figure 1).

### 3.2. Genetic Variation

**Figure 2:**
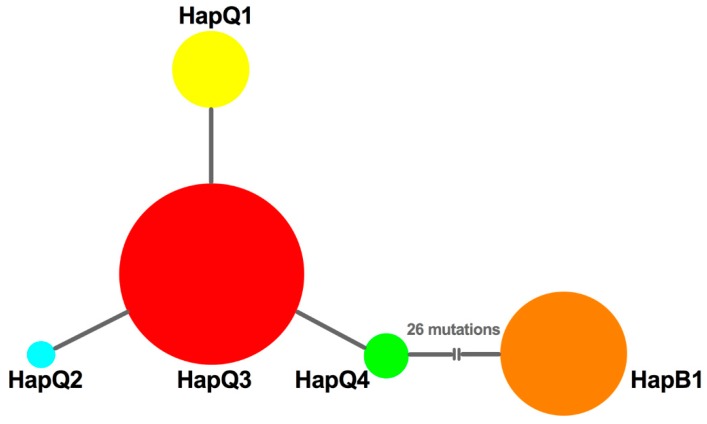
The network of haplotypes of *B. tabaci*. The sizes of circles are proportional to the number of individuals. HapQ1, HapQ2, HapQ3, and HapQ4 are haplotypes of MED. HapB1 is a haplotype of MEAM1.

To decipher the possible genetic mechanisms underlying the successful replacement of MED, we further analyzed the haplotype composition and genetic variation in MEAM1 and MED populations. First, no frameshift mutations or nonsense codons were detected, and the protein sequences could be found in GenBank (accession numbers: KF142153-KF142157). Based on alignment of mtCO1sequences, we found that only one haplotype (named HapB1) was identified from MEAM1 individuals; however, four haplotypes (named HapQ1, HapQ2, HapQ3, and HapQ4) could be defined from MED populations. Of these haplotypes, HapQ3 was the most widely distributed haplotype and was shared among all the populations; HapQ1 was present in all populations except the Wuxi population; HapQ2 and HapQ4 were only detected with low numbers in the Yangzhou and Nantong populations, respectively. Taken together, all populations had at least two haplotypes, except Wuxi. In reference to the time period, HapQ3 was first detected in 2005 from all the populations, except Lianyungang and Suqian; only MEAM1 individuals were found in these two populations in 2005 and 2006; HapQ1 was first detected from Yancheng and Nantong in 2005; HapQ2 and HapQ4 were first detected from the Yangzhou and Nantong populations in 2007 and 2006, respectively. The temporal and spatial distribution of each haplotype can be viewed in Figure S1. Moreover, based on the median joining network of the four MED haplotypes, we found that the network was generally star-like with HapQ1, HapQ2, and HapQ4 as shared mutations from HapQ3 (Figure 2).

The six MED populations showed different levels of genetic variation based on the genetic indexes of H and π. Although the overall level of genetic diversity between MED populations was low, Nantong and Yancheng had higher haplotype diversity (0.352 and 0.285) in comparison to the other four populations. In addition, Nantong had the highest nucleotide diversity, whereas the genetic diversity of Lianyungang and Wuxi were much lower (Table 1).

### 3.3. Population Genetic Structure of MED

**Figure 3:**
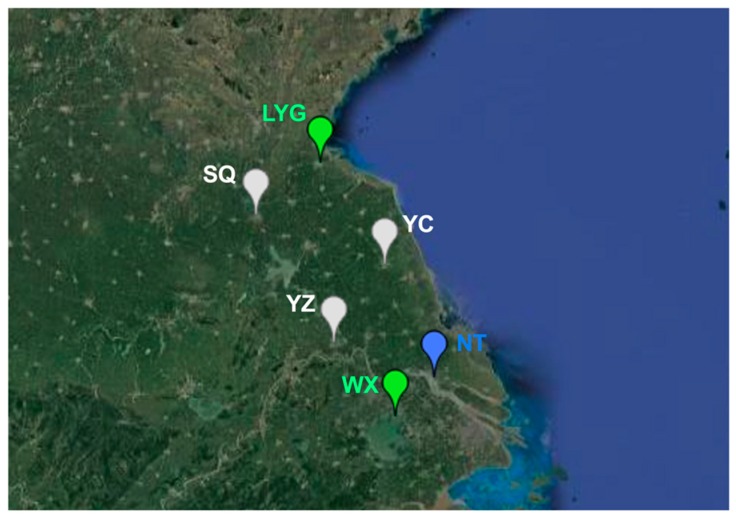
Population genetic structure of *B. tabaci* MED populations inferred by SAMOVA 2.0; LYG: Lianyungang; SQ: Suqian; YC: Yancheng; YZ: Yangzhou; NT: Nantong; WX: Wuxi.

We further investigated the population genetic structure of MED populations. Monitoring of *F*_CT_ values from the SAMOVA analyses suggested three was the optimal number of population groups (*F*_CT3_ = 0.05990; *p* < 0.05). The six populations were clustered into the three groups as follows: (1) Lianyungang and Wuxi, (2) Suqian, Yancheng and Yangzhou, and (3) Nantong (Figure 3). Interestingly, Lianyungang and Wuxi clustered together despite the distance between them being the farthest. The pairwise *F*_ST_ analysis for genetic differentiation between populations confirmed the above clusters. Lianyungang had the lowest genetic differentiation with Wuxi (*F*_ST_ = 0.00370). Likewise, the genetic differentiation between Suqian, Yancheng, and Yangzhou was also low. However, the genetic differentiation between Suqian and Wuxi was the highest (*F*_ST_ = 0.13861) (Table 2).

### 3.4. Neutrality Test and Bayesian Skyline Plot

**Figure 4:**
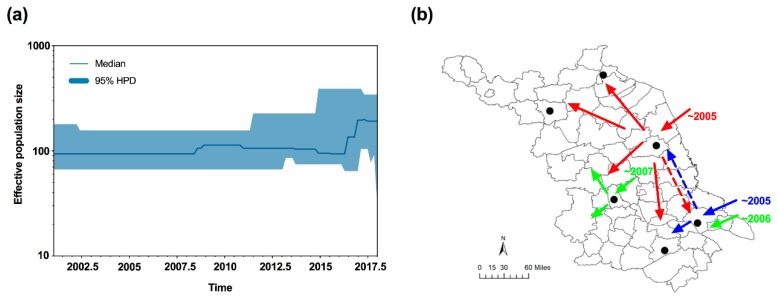
Bayesian skyline plots and inferred invasive routes of *B. tabaci* MED in Jiangsu. (**a**) Bayesian skyline plots of *B. tabaci* populations in Jiangsu. The middle lines represent the median estimates of the effective population size, and the shaded areas represent 95% Highest Posterior Densities (95% HPD). (**b**) Inferred invasive routes of *B. tabaci*. Red and blue arrows indicate the initial invasion routes. Green arrows showed the secondary and/or subsequent invasive events. Dotted lines represented the potential mixing of whiteflies between Nantong and Yancheng. The map of Jiangsu was created using Esri’s ArcGIS platform (http://www.esri.com/software/arcgis).

To uncover the demographic history of MED in Jiangsu, neutrality tests were conducted using Tajima’s *D* and Fu’s *Fs* statistics. The values of Tajima’s *D* and Fu’s *Fs* for all samples were negative (*D* = −0.81772; *Fs* = −1.67605), but not significant (*p* > 0.05); however, a sudden expansion model with a small and insignificant Harpending’s Raggedness (HR) index failed to be rejected (HR = 0.323, *p* = 0.628), which indicated a relatively good fit to a model of demographic expansion [42] or a recent range expansion [43,44]. Furthermore, the median joining network was generally star-like, further confirming the recent population expansion of MED (Figure 2). Importantly, the effective population size estimated by Bayesian skyline plots for the entire MED group exhibited expansion of the population size, representing two recent demographic and range expansion events, around 2007 and 2016, respectively (Figure 4a).

### 3.5. Phylogenetic Reconstruction

We further explored the possible source of MED individuals in Jiangsu based on phylogenetic construction. Based on the constructed phylogenetic tree (Figure S2), four haplotypes of MED individuals mainly clustered into the MED(Q)1 clade, mainly from the western Mediterranean, such as France and Morocco.

## 4. Discussion

The ongoing dispersal of exotic species or the rearrangement of species’ geographical distribution is one of the most striking biological outcomes of global changes [1,45]. In the past decades, two invasive *B. tabaci* species, MEAM1 and MED, were introduced to numerous countries or regions, resulting in large yield loss among different crops. In China, MEAM1 appeared to displace the indigenous whiteflies since it invaded China in the mid-1990s [14]. Interestingly, the introduction of MED to China displaced MEAM1 and other whitefly species over the last decade [46,47]. In this study, we monitored the dynamics of whitefly cryptic species in Jiangsu over 12 years. We discovered that in just three years (from 2005 to 2008), MED almost completely dominated over MEAM1 in most regions. Similarly, Guo et al. reported that MED was first found in Hubei and Shanxi provinces in 2005, and MED had become dominant in several provinces of China by 2007 [48]. In a short time span, the introduction of the MED population excelled in its quest for species establishment. As previously stated, other factors, such as insecticide resistance and host plants, were also hypothesized to contribute to a successful invasion and rapid displacement over a native population. In addition, the factor of genetic traits must also be considered [26,27]. Importantly, since individual level genetic traits have proven to be weak predictors of colonization success, much thought should be given to the population level genetic traits, which have profound influences on the relative success or failure of a colonization event [28]. Therefore, we hypothesized that it was probable that the genetic traits at the population level contributed to the competitive displacement between these two invasive whiteflies.

We first evaluated the genetic variation of a large number of whitefly individuals. As expected, MED populations had a higher level of genetic variation than MEAM1. In particular, only one mitochondrial haplotype could be detected from MEAM1 individuals, whereas four haplotypes of MED were defined; therefore, the overall MED level of haplotype diversity and of nucleotide diversity were much higher than MEAM1. Previous studies had investigated the genetic diversity of MED populations in China [25,48,49,50] and showed that due to bottlenecks or founder effects, the haplotype diversity of MED was lower compared to its presumed Mediterranean origin. However, even though MEAM1 invaded Jiangsu much earlier, MED expressed higher genetic variation than that of MEAM1. Theoretically, a greater diversity should lead to greater adaptability of insects. However, in the present study, we could not make a conclusion that the competitive displacement was a result of the increased genetic diversity of MED. The elevated genetic diversity in the MED was actually a gradual increase in genetic diversity as subsequent haplotypes (HapQ2 and HapQ4) and presumably new populations invaded the province. Half of the locations sampled were predominantly composed of MED haplotypes HapQ3 and HapQ1 in Yencheng and Nantong in 2005 prior to the detection of Q2 and Q4 haplotypes in Yangzhou and Nantong in 2007 and 2006, respectively. The HapQ2 and HapQ4 haplotypes have not spread to other areas in the years since their detection, suggesting that the increased genetic diversity as a result of their presence has not contributed to the displacement of MEAM1. Thus, it would appear that the displacement of the MEAM1 was occurring even from the onset of the invasion when haplotype diversity was low. In some cases, the low genetic diversity might limit the capacity of species to establish in new environments and subsequently spread. However, this does not seem to happen with invasive species that, with little or no genetic variability, manage to have a huge adaptive success. Indeed, numerous invasive populations still thrive and evolve despite the presumed loss of diversity. For example, the beach daisy, *Arctotheca populifolia*, adapted to new environments with very little neutral genetic diversity [51]. In addition, the introduced species such as water hyacinth (*Eichhornia crassipes*) and sweet briar (*Rosa rubiginosa*) are also successful invaders with low neutral genetic diversity [52,53].

Furthermore, it is well known that multiple invasion events by a species have been proposed as a means through which the loss of genetic diversity is overcome [26,54]. In some cases, this has been explained by high levels of propagule pressure through multiple introduction events, resulting in a successful introduction [55,56]. In our study, the multiple invasion events by MED were evaluated by investigating its population genetic structure.

First, we noted that the initial percentages of MED in whitefly populations of the mid-southern part were much higher than those in the northern regions (Suqian and Lianyungang). This potentially indicated that MED could have invaded Jiangsu starting from the mid-southern region. Indeed, MED could not be found from Lianyungang and Suqian until 2007. Furthermore, based on the index of genetic diversity and the number of haplotypes, we could easily find that MED individuals in Yancheng and Nantong had the highest level of genetic diversity. Because “ancestral” populations typically possess higher levels of genetic diversity [57,58,59], we speculated that the other MED populations in Jiangsu originated from Yancheng or Nantong. Indeed, the field observation and population genetic structure confirmed the multiple introduction sites. Three MED genetic groups were identified, and individuals from Lianyungang and Wuxi were clustered in the same group despite the extreme distance. The most plausible explanation is that MED individuals in Lianyungang and Wuxi originated from Yancheng or Nantong, which are two regions located between them. Of note, HapQ4 was only found in Nantong at a relatively high percentage. This could indicate that Nantong might be one of the introduction sites in Jiangsu. Taken together, MED probably invaded Jiangsu separately starting from Yancheng or Nantong.

Second, due to the high population densities of whiteflies and frequent agricultural trading in Jiangsu, secondary and/or subsequent invasions of MED to Jiangsu are possible. Previous studies demonstrated that MED initially invaded and established populations in the Yangtze River Valley and eastern coastal areas [11,13,48], allowing for multiple introductions of MED in Jiangsu. Our analyses of haplotypes, genetic diversity, and genetic structure of MED confirmed the hypothesis of multiple invasion events. First, the new haplotypes of HapQ2 and HapQ4 were detected in Yangzhou and Nantong in 2007 and 2006, respectively, which occurred after the initial detection of MED in Jiangsu. In addition, the effective population size estimated by the Bayesian skyline plots demonstrated two recent demographic and range expansion events, which occurred in 2007 and 2016. For the cluster group of Lianyugang and Wuxi, the individuals from this group were validated to be from the central part. Since the initial invasive event was fairly recent, all populations should be clustered as the same group; however, three groups were identified. The most plausible explanation could be that there were secondary or subsequently more invasive events in the central region, and these events have not yet occurred in the northern part of Jiangsu. In addition, based on the genetic diversity analyses of six populations, we speculated that MED formed the bridgehead populations in Yancheng and Nantong, where it had the higher genetic diversity. Under a bridgehead scenario, a successfully established invasive population can serve as a source of colonists for new invasions and thereby give rise to secondary and/or subsequent introductions [60].

To some extent, the Yangtze River probably acted as a physical barrier for MED, because all populations had the haplotype HapQ1 except Wuxi, which is the only population located in the southern part of the Yangtze River. We suspect that Wuxi could be one of the introduction sites. Furthermore, the reason why MED in Wuxi did not successfully displace MEAM1 could be because MED had only one haplotype and expressed the lowest level of genetic diversity. This may support our idea concerning the contribution of genetic traits in successful invasion and displacement.

Finally, we utilized phylogenetic construction to explore the possible source for MED individuals in Jiangsu. The MED individuals can be separated into two subclades, MED(Q)1 and MED(Q)2, from the Mediterranean [61]. In our study, four haplotypes of MED individuals mainly clustered into the MED(Q)1 clade, which was mainly from the western Mediterranean such as France and Morocco. Similarly, MED individuals in Shandong Province were also grouped into subclade MED(Q)1 [25,50]. Therefore, MED populations in these two regions may have been introduced from the same invasion source.

## 5. Conclusions

In summary, we monitored the invasion of MED populations along with the rapid displacement of the MEAM1 populations in Jiangsu over a 12-year period. We gave evidence that the multiple invasions of MED occurred in Jiangsu based on our monitoring and genetic data. Importantly, the multiple introductions probably enhanced the ability of MED populations to occupy Jiangsu and dominate over MEAM1 by accelerating its geographic expansion. Finally, we inferred the invasive routes of *B. tabaci* MED in Jiangsu. Specifically, MED first invaded Jiangsu separately starting from either Nantong or Yancheng in 2005, then formed bridgehead populations, and proceeded to invade other regions in Jiangsu. In addition, from 2005 to 2007, there were probably secondary and/or subsequent invasive events into Yangzhou and Nantong (Figure 4b). The combination of field survey and genetic data helped us not only uncover the process of a successful whitefly’s invasion, but also offer new perspectives for the mechanism responsible for the displacement of one population over another. However, in the present study, we did not consider the effects of host plants. Overall, our study provided a further explanation of the successful invasion and rapid displacement between the two invasive whitefly species.

## Figures and Tables

**Table 1:** Parameters of genetic diversity of six *B. tabaci* MED populations.

Genetic Index	Lianyungang	Suqian	Yancheng	Yangzhou	Nantong	Wuxi
Haplotype Diversity (H)	0.080	0.220	0.285	0.215	0.352	0.000
Nucleotide Diversity (π)	0.00014	0.00039	0.00039	0.00039	0.00078	0.00000

**Table 2:** Pairwise estimates of *F*_ST_ between six populations of *B. tabaci* MED populations.

Populations	Lianyungang	Suqian	Yancheng	Yangzhou	Nantong	Wuxi
Lianyungang		−	−	−	−	−
Suqian	0.02640		−	−	−	−
Yancheng	0.03546	−0.03945		−	−	+
Yangzhou	0.01216	−0.02506	−0.00666		−	−
Nantong	0.02413	0.01178	0.03126	0.02137		+
Wuxi	0.00370	0.13861	0.10941	0.04131	0.05545	

Values below the diagonal represent *F*_ST_ values; symbols above the diagonal are the significance of *F*_ST_. “+” represents significance and “−” represents non-significance (significance level = 0.05).

## Supplementary Materials

The following are available online at https://www.mdpi.com/2075-4450/11/1/35/s1: Figure S1: Temporal and spatial distribution of each haplotype of MED. Figure S2: Phylogenetic tree of mtCO1 sequences for *B. tabaci*. Trees are from Bayesian Inferences (BI, left) and Maximum Likelihood analyses (ML, right). Numbers at the nodes are the posterior probabilities as support values. The greenhouse whitefly *Trialeurodes vaporariorum* was used as the outgroup. ●: sequences of different haplotypes of *B. tabaci* obtained in this paper. Table S1: Collection details and sequenced samples of *B. tabaci* in Jiangsu Province from 2005 to 2016.

## References

[1] Gao Y., Reitz S.R. (2017). Emerging themes in our understanding of species displacements. Annu. Rev. Entomol..

[2] De Barro P.J., Liu S.-S., Boykin L.M., Dinsdale A.B. (2011). *Bemisia tabaci*: A statement of species status. Annu. Rev. Entomol..

[3] Dinsdale A., Cook L., Riginos C., Buckley Y., De Barro P. (2010). Refined global analysis of *Bemisia tabaci* (Hemiptera: Sternorrhyncha: Aleyrodoidea: Aleyrodidae) mitochondrial cytochrome oxidase 1 to identify species level genetic boundaries. Ann. Entomol. Soc. Am..

[4] Hu J., Zhang X., Jiang Z., Zhang F., Liu Y., Li Z., Zhang Z. (2018). New putative cryptic species detection and genetic network analysis of *Bemisia tabaci* (Hempitera: Aleyrodidae) in China based on mitochondrial COI sequences. Mitochondrial DNA Part A.

[5] Wang X.W., Li P., Liu S.S. (2017). Whitefly interactions with plants. Curr. Opin. Insect Sci..

[6] Boykin L.M., Armstrong K.F., Kubatko L., De Barro P. (2012). Species delimitation and global biosecurity. Evol. Bioinform..

[7] Chu D., Zhang Y., Cong B., Xu B., Wu Q. (2005). Identification for Yunnan Q-biotype *Bemisia tabaci* population. Entomol. Knowl..

[8] Chu D., Zhang Y.-J., Brown J.K., Cong B., Xu B.-Y., Wu Q.-J., Zhu G.-R. (2006). The introduction of the exotic Q biotype of *Bemisia tabaci* from the Mediterranean region into China on ornamental crops. Fla. Entomol..

[9] Teng X., Wan F.-H., Chu D. (2010). *Bemisia tabaci* biotype Q dominates other biotypes across China. Fla. Entomol..

[10] Wang Z., Yan H., Yang Y., Wu Y. (2010). Biotype and insecticide resistance status of the whitefly *Bemisia tabaci* from China. Pest Manag. Sci..

[11] Hu J., De Barro P., Zhao H., Wang J., Nardi F., Liu S.-S. (2011). An extensive field survey combined with a phylogenetic analysis reveals rapid and widespread invasion of two alien whiteflies in China. PLoS ONE.

[12] Pan H., Chu D., Ge D., Wang S., Wu Q., Xie W., Jiao X., Liu B., Yang X., Yang N. (2011). Further spread of and domination by *Bemisia tabaci* (Hemiptera: Aleyrodidae) biotype Q on field crops in China. J. Econ. Entomol..

[13] Rao Q., Luo C., Zhang H., Guo X., Devine G. (2011). Distribution and dynamics of *Bemisia tabaci* invasive biotypes in central China. Bull. Entomol. Res..

[14] Liu S.-S., De Barro P., Xu J., Luan J.-B., Zang L.-S., Ruan Y.-M., Wan F.-H. (2007). Asymmetric mating interactions drive widespread invasion and displacement in a whitefly. Science.

[15] Liu B., Yan F., Chu D., Pan H., Jiao X., Xie W., Wu Q., Wang S., Xu B., Zhou X. (2012). Difference in feeding behaviors of two invasive whiteflies on host plants with different suitability: Implication for competitive displacement. Int. J. Biol. Sci..

[16] Sun D.-B., Li J., Liu Y.-Q., Crowder D.W., Liu S.-S. (2014). Effects of reproductive interference on the competitive displacement between two invasive whiteflies. Bull. Entomol. Res..

[17] Luo C., Jones C., Devine G., Zhang F., Denholm I., Gorman K. (2010). Insecticide resistance in *Bemisia tabaci* biotype Q (Hemiptera: Aleyrodidae) from China. Crop Prot..

[18] Sun D.-B., Liu Y.-Q., Qin L., Xu J., Li F.-F., Liu S.-S. (2013). Competitive displacement between two invasive whiteflies: Insecticide application and host plant effects. Bull. Entomol. Res..

[19] De Barro P., Bourne A. (2010). Ovipositional host choice by an invader accelerates displacement of its indigenous competitor. Biol. Invasions.

[20] De Barro P., Bourne A., Khan S., Brancatini V. (2006). Host plant and biotype density interactions–their role in the establishment of the invasive B biotype of *Bemisia tabaci*. Biol. Invasions.

[21] Chu D., Tao Y.L., Zhang Y.J., Wan F.H., Brown J.K. (2012). Effects of host, temperature and relative humidity on competitive displacement of two invasive *Bemisia tabaci* biotypes [Q and B]. Insect Sci..

[22] Xiao N., Pan L.-L., Zhang C.-R., Shan H.-W., Liu S.-S. (2016). Differential tolerance capacity to unfavourable low and high temperatures between two invasive whiteflies. Sci. Rep..

[23] Jiu M., Zhou X.-P., Tong L., Xu J., Yang X., Wan F.-H., Liu S.-S. (2007). Vector-virus mutualism accelerates population increase of an invasive whitefly. PLoS ONE.

[24] Liu J., Zhao H., Jiang K., Zhou X.P., Liu S.S. (2009). Differential indirect effects of two plant viruses on an invasive and an indigenous whitefly vector: Implications for competitive displacement. Ann. Appl. Biol..

[25] Li H.-R., Pan H.-P., Tao Y.-L., Zhang Y.-J., Chu D. (2017). Population genetics of an alien whitefly in China: Implications for its dispersal and invasion success. Sci. Rep..

[26] Miura O. (2007). Molecular genetic approaches to elucidate the ecological and evolutionary issues associated with biological invasions. Ecol. Res..

[27] Tsutsui N.D., Suarez A.V., Holway D.A., Case T.J. (2000). Reduced genetic variation and the success of an invasive species. Proc. Natl. Acad. Sci. USA.

[28] Crawford K., Whitney K. (2010). Population genetic diversity influences colonization success. Mol. Ecol..

[29] Shen Y., Du Y.-Z., Ren S., Qiu B. (2011). Preliminary study of succession of *Bemisia tabaci* biotypes in Jiangsu Province, China. Chin. J. Appl. Entomol..

[30] Luo C., Yao Y., Wang R., Yan F., Hu D., Zhang Z. (2002). The use of mitochondrial cytochrome oxidase I (mt CO I) gene sequences for the identification of biotypes of *Bemisia tabaci* (Gennadius) in China. Acta Entomol. Sin..

[31] Shatters R.G., Powell C.A., Boykin L.M., Liansheng H., McKenzie C. (2009). Improved DNA barcoding method for *Bemisia tabaci* and related Aleyrodidae: Development of universal and *Bemisia tabaci* biotype-specific mitochondrial cytochrome c oxidase I polymerase chain reaction primers. J. Econ. Entomol..

[32] Chenna R., Sugawara H., Koike T., Lopez R., Gibson T.J., Higgins D.G., Thompson J.D. (2003). Multiple sequence alignment with the Clustal series of programs. Nucleic Acids Res..

[33] Librado P., Rozas J. (2009). DnaSP v5: A software for comprehensive analysis of DNA polymorphism data. Bioinformatics.

[34] Bandelt H.-J., Forster P., Röhl A. (1999). Median-joining networks for inferring intraspecific phylogenies. Mol. Biol. Evol..

[35] Dupanloup I., Schneider S., Excoffier L. (2002). A simulated annealing approach to define the genetic structure of populations. Mol. Ecol..

[36] Excoffier L., Lischer H.E. (2010). Arlequin suite ver 3.5: A new series of programs to perform population genetics analyses under Linux and Windows. Mol. Ecol. Resour..

[37] Huelsenbeck J.P., Ronquist F. (2001). MRBAYES: Bayesian inference of phylogenetic trees. Bioinformatics.

[38] Guindon S., Lethiec F., Duroux P., Gascuel O. (2005). PHYML Online—A web server for fast maximum likelihood-based phylogenetic inference. Nucleic Acids Res..

[39] Drummond A.J., Rambaut A., Shapiro B., Pybus O.G. (2005). Bayesian coalescent inference of past population dynamics from molecular sequences. Mol. Biol. Evol..

[40] Drummond A.J., Rambaut A. (2007). BEAST: Bayesian evolutionary analysis by sampling trees. BMC Evol. Biol..

[41] Rambaut A., Drummond A.J., Xie D., Baele G., Suchard M.A. (2018). Posterior summarisation in Bayesian phylogenetics using Tracer 1.7. Syst. Biol..

[42] Rogers A.R., Harpending H. (1992). Population growth makes waves in the distribution of pairwise genetic differences. Mol. Biol. Evol..

[43] Ray N., Currat M., Excoffier L. (2003). Intra-deme molecular diversity in spatially expanding populations. Mol. Biol. Evol..

[44] Excoffier L. (2004). Patterns of DNA sequence diversity and genetic structure after a range expansion: Lessons from the infinite-island model. Mol. Ecol..

[45] Vitousek P.M., Loope L.L., Westbrooks R. (1996). Biological Invasions As Global Environmental Change.

[46] Chu D., Wan F.H., Zhang Y.J., Brown J.K. (2010). Change in the biotype composition of *Bemisia tabaci* in Shandong Province of China from 2005 to 2008. Environ. Entomol..

[47] Chu D., Zhang Y.J., Wan F.H. (2010). Cryptic invasion of the exotic *Bemisia tabaci* biotype Q occurred widespread in Shandong Province of China. Fla. Entomol..

[48] Guo X.-J., Rao Q., Zhang F., Chen L., Zhang H.-Y., Gao X.-W. (2012). Diversity and genetic differentiation of the whitefly *Bemisia tabaci* species complex in China based on mtCOI and cDNA-AFLP analysis. J. Integr. Agric..

[49] Zhang L., Zhang Y., Zhang W., Wu Q., Xu B., Chu D. (2005). Analysis of genetic diversity among different geographical populations and determination of biotypes of *Bemisia tabaci* in China. J. Appl. Entomol..

[50] Chu D., Gao C., De Barro P., Wan F., Zhang Y. (2011). Investigation of the genetic diversity of an invasive whitefly (*Bemisia tabaci*) in China using both mitochondrial and nuclear DNA markers. Bull. Entomol. Res..

[51] Rollins L.A., Moles A.T., Lam S., Buitenwerf R., Buswell J.M., Brandenburger C.R., Flores-Moreno H., Nielsen K.B., Couchman E., Brown G.S. (2013). High genetic diversity is not essential for successful introduction. Ecol. Evol..

[52] Ren M.X., Zhang Q.G., Zhang D.Y. (2005). Random amplified polymorphic DNA markers reveal low genetic variation and a single dominant genotype in Eichhornia crassipes populations throughout China. Weed Res..

[53] Zimmermann H., Ritz C.M., Hirsch H., Renison D., Wesche K., Hensen I. (2010). Highly reduced genetic diversity of Rosa rubiginosa L. populations in the invasive range. Int. J. Plant Sci..

[54] Frankham R. (2005). Resolving the genetic paradox in invasive species. Heredity.

[55] Kolbe J.J., Glor R.E., Schettino L.R., Lara A.C., Larson A., Losos J.B. (2004). Genetic variation increases during biological invasion by a Cuban lizard. Nature.

[56] Genton B., Shykoff J., Giraud T. (2005). High genetic diversity in French invasive populations of common ragweed, *Ambrosia artemisiifolia*, as a result of multiple sources of introduction. Mol. Ecol..

[57] Savolainen P., Zhang Y.-P., Luo J., Lundeberg J., Leitner T. (2002). Genetic evidence for an East Asian origin of domestic dogs. Science.

[58] Ma C., Yang P., Jiang F., CHAPUIS M.P., Shali Y., Sword G.A., Kang L. (2012). Mitochondrial genomes reveal the global phylogeography and dispersal routes of the migratory locust. Mol. Ecol..

[59] Zhang B., Edwards O., Kang L., Fuller S. (2012). Russian wheat aphids (*Diuraphis noxia*) in China: Native range expansion or recent introduction?. Mol. Ecol..

[60] Bertelsmeier C., Ollier S., Liebhold A.M., Brockerhoff E.G., Ward D., Keller L. (2018). Recurrent bridgehead effects accelerate global alien ant spread. Proc. Natl. Acad. Sci. USA.

[61] Chu D., Wan F.H., Tao Y.L., Liu G.X., Fan Z.X., Bi Y.P. (2008). Genetic differentiation of *Bemisia tabaci* (Gennadius) (Hemiptera: Aleyrodidae) biotype Q based on mitochondrial DNA markers. Insect Sci..

