# Supplementary material for: Competitive Displacement between Bemisia tabaci MEAM1 and MED and Evidence for Multiple Invasions of MED
Source: Insects. 2019 Dec 31;11(1):35. doi: 10.3390/insects11010035 (PMC7022974; doi:10.3390/insects11010035)
Supplement: Supplementary file 1 [file insects-11-00035-s001.pdf]

Table 1. Collection details and sequenced samples of *B. tabaci* in Jiangsu Province from 2005 to 2016.

Year	Location	Host Plant (Common Name)	Species Numbers			
			MED	MEAM1	Asia II 3	Total
**2005**	**Lianyungang**	Cucumber	0	20	0	20
	Suqian	Eggplant, Tomato	0	30	0	30
	Yancheng	Cucumber, Dahlias, Canadian Thistle, Goldenrod, Cole, Eggplant	46	14	0	60
	Yangzhou	Cotton, Sow thistle, Radish, Prickly Sowthistle, Burdock, Tomato	6	63	1	70
	Nantong	Cotton, Tomato, Broccoli, Cucumber, Capsicum, Melon	29	31	0	60
	Wuxi	Japanse hop, Tomato, Capsicum, Lentils	49	11	0	60
2006	Lianyungang	Tomato, Eggplant, Soybean, Cabbage	0	40	0	40
	Suqian	Eggplant, Lentils, Japanese hop	0	30	0	30
	Yancheng	Lentils, Eggplant, Cotton, Cucumber, Capsicum	60	0	0	60
	Yangzhou	Cucumber, Eggplant, Cowpea	9	21	0	30
	Nantong	Eggplant, Capsicum, Cabbage, Tomato, Cucumber, Squash	50	20	0	70
	Wuxi	Tomato, Japanese hop, Cucumber	20	10	0	30
2007	Lianyungang	Tomato, Cucumber, Eggplant	7	23	0	30
	Suqian	Cucumber, Eggplant	25	5	0	30
	Yancheng	Tomato, Japanese hop, Cotton	37	3	0	40
	Yangzhou	Eggplant, Japanese hop, Cucumber, Lentils, Eggplant, Pumpkin	48	2	0	50
	Nantong	Tomato, Cotton, Eggplant, Japanese hop	63	7	0	70
	Wuxi	Tomato, Capsicum, Cucumber	32	8	0	40
2008	Lianyungang	Eggplant, Cotton, Japanese hop	40	10	10	60
	Suqian	Cucumber, Eggplant, Capsicum, Cowpea	31	19	0	50
	Yancheng	Tomato, Capsicum, Cucumber, Eggplant	30	0	0	30
	Yangzhou	Eggplant, Cucumber, Capsicum,	60	0	0	60
	Nantong	Cotton, Eggplant, Yamaimo, Cucumber, Capsicum	75	0	0	75
	Wuxi	Capsicum, Cucumber, Broccoli	24	16	10	50
2009	Lianyungang	Eggplant, Pumpkin, Cotton, Japanese hop, Cucumber	48	2	0	50
	Suqian	Lentils, Tomato, Capsicum, Lentils	33	2	0	35
	Yancheng	Capsicum, Eggplant, Cotton, Cucumber	40	0	0	40
	Yangzhou	Mustard, Eggplant, Capsicum, Cucumber, Gerbera	60	0	0	60
	Nantong	Cucumber, Eggplant, Melon	59	1	0	60
	Wuxi	Cucumber, Tomato, Capsicum, Eggplant	43	27	0	70
2010	Lianyungang	Eggplant, Piemarke	20	0	0	20
	Suqian	Soybean, Eggplant	20	0	0	20
	Yancheng	Eggplant, Cotton, Tomato	30	0	0	30
	Yangzhou	Capsicum, Tomato, Lentils	50	0	0	50
	Nantong	Cucumber, Tomato, Eggplant	30	0	0	30
	Wuxi	Cucumber, Eggplant, Pumpkin, Piemarker	31	29	0	60
2011	Lianyungang	Piemarker, Cucumber, Eggplant, Pumpkin	46	4	0	50
	Suqian	Eggplant, Pumpkin	20	0	0	20
	Yancheng	Capsicum, Eggplant, Cowpea, Cotton	70	0	0	70
	Yangzhou	Soybean, Tomato, Eggplant, Cowpea, Capsicum, Cucumber	60	0	0	60
	Nantong	Capsicum, Lettuce, Broccoli, Eggplant, Tomato	60	0	0	60
	Wuxi	Eggplant, Radish, Pumpkin	21	19	0	40
2012	Lianyungang	Soybean, Cucumber, Lentils, Eggplant, Pumpkin	60	0	0	60
	Suqian	Eggplant, Sweet potato, Melon, Tomato, Pumpkin, Cucumber	59	1	0	60
	Yancheng	Eggplant, Lentils, Cucumber	30	0	0	30
	Yangzhou	Eggplant, Pumpkin, Melon, Sunflower, Cucumber	60	0	0	60
	Nantong	Cucumber, Tomato, Eggplant, Capsicum	60	0	0	60
	Wuxi	Cucumber, Pakchoi, Pumpkin, Cabbage, Pumpkin	30	30	0	60
2013	Lianyungang	Urtica, Cucumber, Eggplant, Pumpkin	60	0	0	60
	Suqian	Soybean, Pumpkin, Japanese hop, Pepper	79	1	0	80
	Yancheng	Soybean, tomato, Pumpkin, Japanese hop, Eggplant, Cotton, Prpper, Pumpkin, Cabbage	130	0	0	130
	Yangzhou	Japanese hop, Pumpkin, Cabbage, Pepper	70	0	0	70
	Nantong	Soybean, Pumpkin, Pepper, Eggplant, Tomato, Cotton, Cabbage	150	0	0	150
	Wuxi	Cucumber, Tomato, Eggplant, Lettuce	42	48	0	90
2014	Lianyungang	Japanese hop, Pumpkin, Cabbage, Pepper, Eggplant, Tomato	106	2	0	108

	Suqian	Pumpkin, Sweet potato, Melon	82	2	0	84
	Yancheng	Pumpkin, Eggplant, Pepper, Cucumber	120	0	0	120
	Yangzhou	Pumpkin, Eggplant, Pepper, Cucumber, Tomato, Japanese hop	96	0	0	96
	Nantong	Pumpkin, Sweet potato, Melon, Pepper, Eggplant, Tomato	129	3	0	132
	Wuxi	Cucumber, Tomato, Eggplant, Lettuce, Pepper, Cabbage	66	78	0	144
2015	Lianyungang	Japanese hop, Pumpkin, Eggplant, Pepper, Cucumber	119	1	0	120
	Suqian	Cucumber, Eggplant, Lettuce, Sweet potato, Japanese hop	82	2	0	84
	Yancheng	Eggplant, Tomato, Pumpkin, Cucumber, Japanese hop, Cabbage	156	0	0	156
	Yangzhou	Japanese hop, Pumpkin, Cabbage, Pepper, Eggplant, Tomato, Cowpea	120	0	0	120
	Nantong	Eggplant, Lentils, Cucumber, Toamto, Cotton, Japanese hop, Pepper	127	35	0	162
	Wuxi	Tomato, Cowpea, Cucumber, Lettuce	37	11	0	48
2016	Lianyungang	Pumpkin, Sweet potato, Cucumber, Pepper, Eggplant, Tomato	162	0	0	162
	Suqian	Eggplant, Tomato, Pumpkin, Cucumber, Japanese hop	119	1	0	120
	Yancheng	Eggplant, Sweet potato, Melon, Tomato, Pumpkin, Cucumber	114	0	0	114
	Yangzhou	Cucumber, Eggplant, Pepper, Japanese hop, Pumpkin	111	1	0	112
	Nantong	Cucumber, Eggplant, Pepper, Japanese hop, Pumpkin, Sweet potato,	120	0	0	120
	Wuxi	Eggplant, Cucumber, Cowpea, Sweet potato, Cabbage	23	31	0	54

MED (Mediterranean), MEAM1 (Middle East-Asia Minor 1) and Asia II 3 are three whitefly cryptic species.

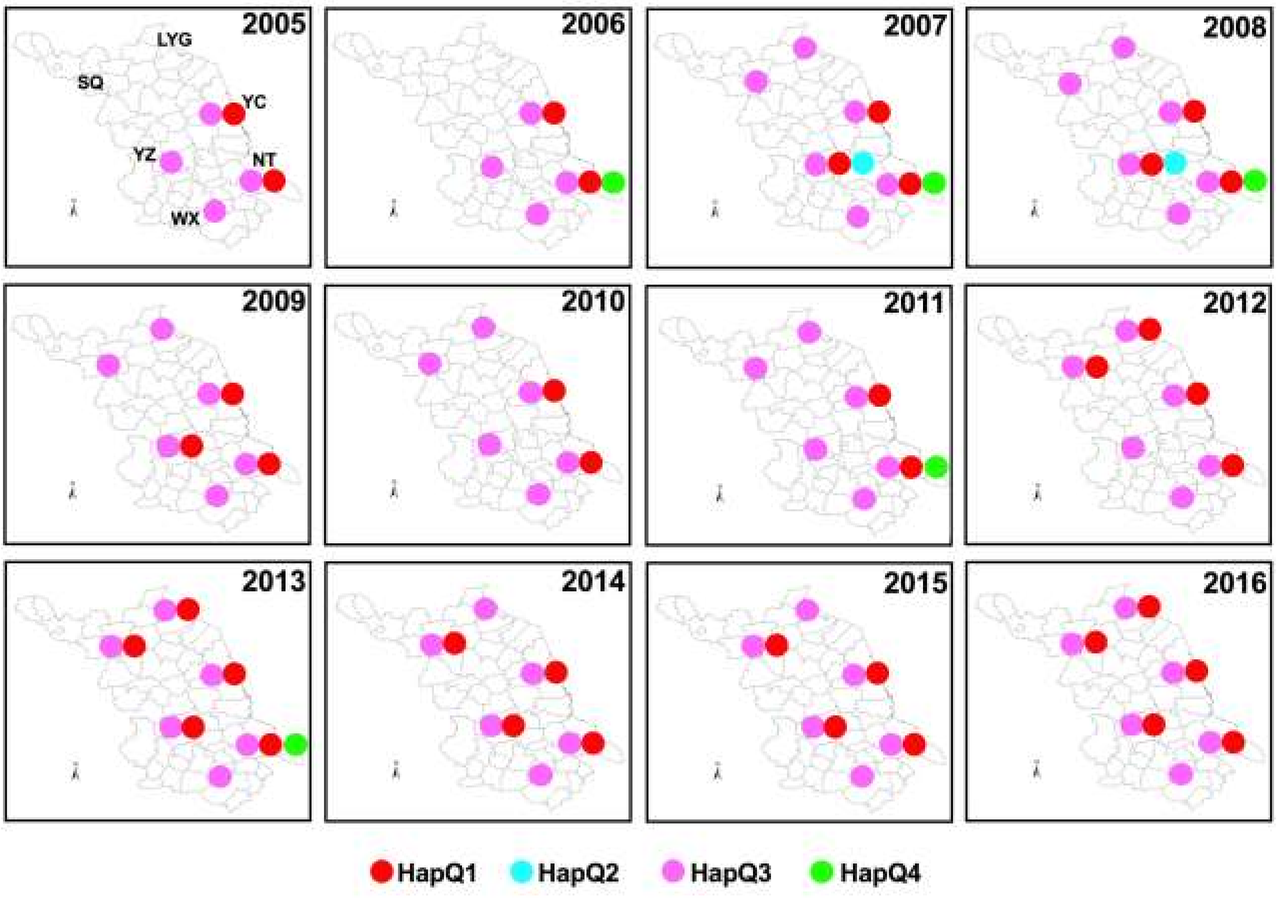

Figure S1. Temporal and spatial distribution of each haplotype of MED.

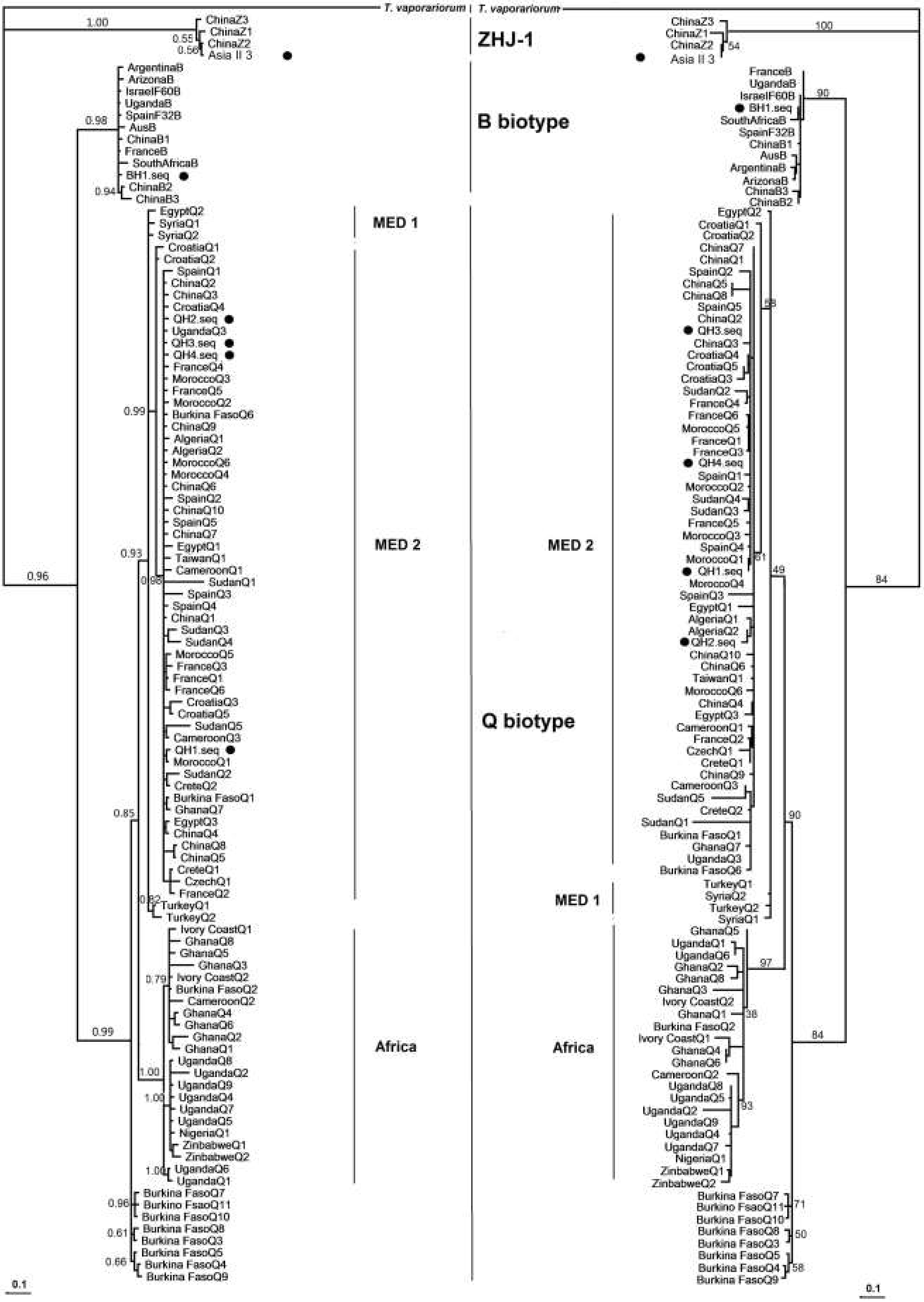

Figure S2. Phylogenetic tree of mtCO1 sequences for *B. tabaci.* Trees are from Bayesian inferences (BI, left) and maximum-likelihood analyses (ML, right). Numbers at the nodes are the posterior probabilities as support values. The greenhouse whitefly *Trialeurodes vaporariorum* was used as an outgroup. ●: sequences of different haplotypes of *B. tabaci* obtained in this paper.
